# The expression of cytokines in the milk somatic cells, blood leukocytes and serum of goats infected with small ruminant lentivirus

**DOI:** 10.1186/s12917-019-2182-4

**Published:** 2019-11-27

**Authors:** Justyna Jarczak, Danuta Słoniewska, Jarosław Kaba, Emilia Bagnicka

**Affiliations:** 1 Biobank Lab, Department of Molecular Biophysics, University of Łódź, ul. Pilarskiego 14/16, 90-231 Łódź, Poland; 2 Polish Center for Technology Development – PORT, BBMRI.pl Consortium, ul. Stabłowicka 147, 54-066 Wrocław, Poland; 3 Institute of Genetics and Animal Breeding, Polish Academy of Sciences, Jastrzębiec, ul. Postępu 36A, 05-552 Magdalenka, Poland; 4 Division of Epidemiology and Veterinary Economics, Institute of Veterinary Medicine, Warsaw University of Life Sciences, ul. Nowoursynowska 159c, 02-787 Warszawa, Poland

**Keywords:** Goats, SRLV, Gene expression, Protein expression

## Abstract

**Background:**

The present study aimed to determine the expression of cytokines, which is associated with the immunological response of dairy goats against small ruminant lentivirus (SRLV). The study was conducted on 26 dairy goats in their second to sixth lactation, which were divided by breed and parity into two groups: SRLV naturally infected (*N* = 13) and non-infected (N = 13) animals. All goats in the study were asymptomatic. The milk and blood samples, which served as studied material were taken on days 7, 30, 120 and 240 of the lactation. The gene and protein expression of several cytokines was studied using Real-Time PCR and ELISA methods.

**Results:**

*INF-β* and *INF-γ* expression was down-regulated in the milk somatic cells (MSC) of SRLV-infected goats. However, an increased concentration of INF-β was observed in the MSC in SRLV-infected goats, while INF-γ expression was not observed in both SRLV-infected and non-infected animals The SRLV-infected goats also displayed decreased expression of *IL-1α, IL-1β*, *IL-6* and *INF-γ* genes in the blood leukocytes,with IL-1α, IL-1β and IL-6 protein levels also being decreased in the sera. *TNF-α* was the only gene that demonstrated increased expression in both the MSC and the blood of infected animals; however, no such overexpression was observed at the protein level.

**Conclusions:**

SRLV probably influences the immune system of infected animals by deregulating of the expression of cytokines. Further, epigenetic studies may clarify the mechanisms by which SRLV regulates the gene and protein expression of the host.

## Background

As members of *Retroviridae* family and *Lentivirinae* subfamily, small ruminant lentivirus (SRLV) is related to equine (EIAV), feline (FIV), simian (SIV) and human (HIV) immunodeficiency viruses, and is one of the most common pathogens in goat herds, causing long-lasting disease in the mammary gland and joints. The virus is known to cause chronic inflammation of various tissues and organs, and although it does not cause immune deficiency, infection is known to influence the functioning of the immune system [1]. It mainly infects the cells of the innate immune system, such as macrophages, monocytes and dendritic cells, but not lymphocytes. It can also infect the central nervous system (CNS), however, such cases are rare and are only observed in very young animals, with immediate lethal effect [2]. Although infection can develop for many years without clinical signs, no specific therapy exists for infected animals, nor any vaccine for the prevention of SRLV infection [3].

Viral infections may deregulate immune responses [4], and as such, there is a great need for studies to recognize and understand the host immune responses against these pathogens. Following SRLV infection, the main producers of cytokines associated with the early response are macrophages and monocytes [4]. A key role in the cellular response to pathogens is played by macrophages: antigen-presenting cells that produce cytokines such as interferon gamma (IFNγ) [5], interleukin-1 alpha (IL-1α), and tumor necrosis factor alpha (TNFα) [6, 7], which are essential components of the antiviral immune defense. Lechner et al. [8] studied the impact of SRLV on the expression of cytokines in macrophages and the importance of deregulated cytokine responses. Using in situ hybridization and RNA blot slot analysis it was found that infection of macrophages by SRLV increased the expression of interleukin 8 (IL-8) and monocyte chemoattractant protein 1 (MCP-1) compared to uninfected cells. In addition interleukin 16 (IL-16), a proinflammatory cytokine produced by macrophages, among others, displays increased expression (mRNA and protein) in the blood of SRLV-infected goats; the authors suggested that increased IL-16 expression during SRLV infection may inhibit viral integration. In another study [9], the expression of the gamma interferon (*IFN-γ*), interleukin 2 (*IL-2*) and interleukin 4 (*IL-4*) genes of peripheral blood mononuclear cells (PBMC) obtained from Saanen goats was evaluated by semi quantitative reverse transcriptase PCR; the goats had previously been experimentally infected with gp135 surface protein (SU) purified from SRLV [9]. While IFN-γ and IL-4 expression of the SU-responsive PBMC cells showed differences in lymphokine balance, which was associated with the disease status between asymptomatic and arthritic, no such change in *IL-2* gene expression was observed .

Hence, further studies are needed to better understand the immune system of goats infected with SRLV, especially those aspects related to gene and protein expression. The differences found between infected and non-infected animals can highlight changes in the inflammation process and clarify the deregulation of the immune response. The aim of the present study is, hence, to identify the cytokines associated with the immunological response against SRLV; to this end, it determines the expression of cytokines in milk somatic cells at the mRNA and protein levels, and in the blood leukocytes (mRNA) and serum (protein) of non-infected and SRLV-infected goats.

## Results

### Cytokine expression at the transcript level in milk somatic cells and blood leukocytes

**Fig. 1:**
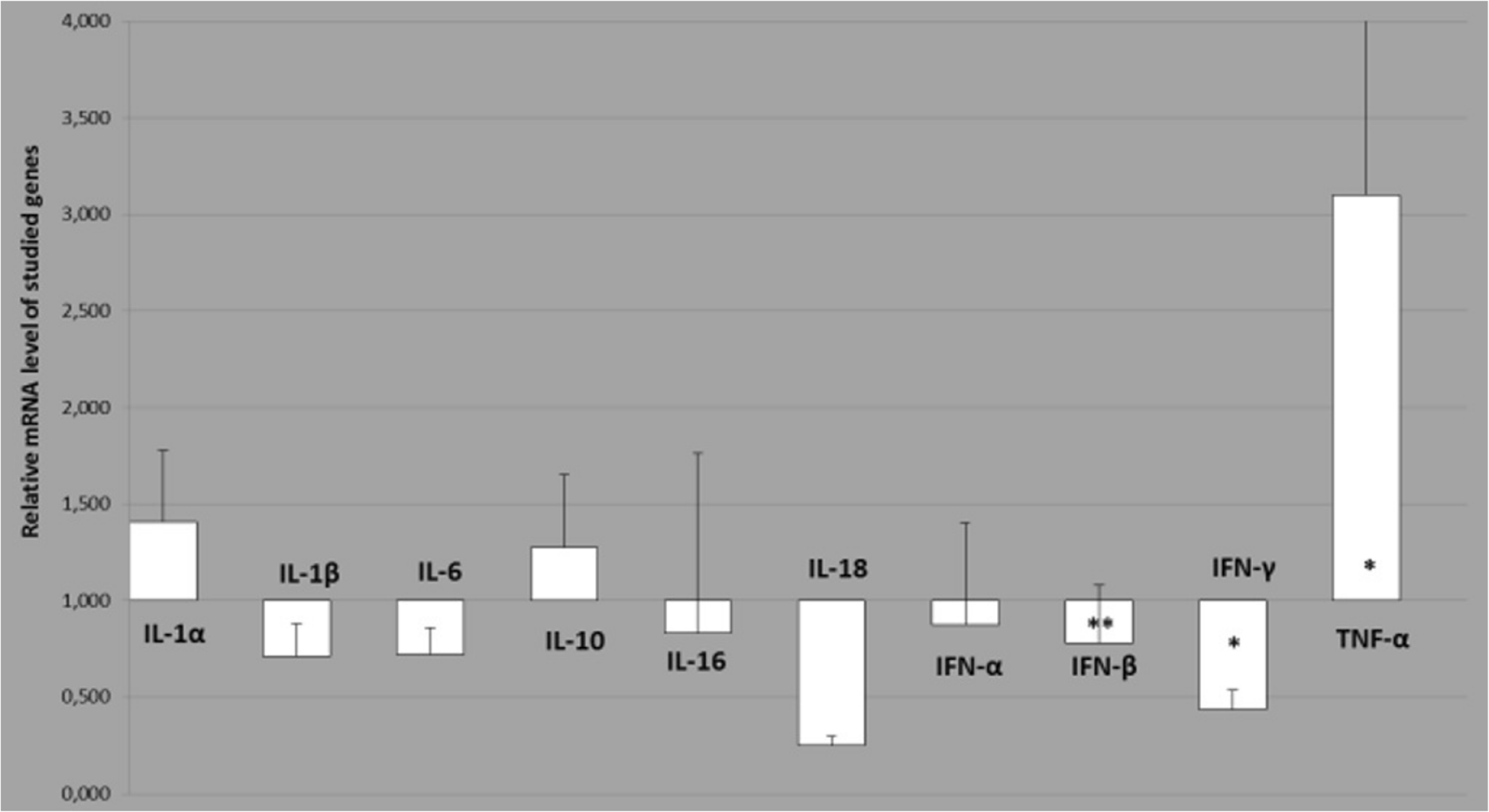
Relative expression of cytokine genes in the MSC of SRLV-infected animals comparing to non-infected ones

No differences in gene expression were observed between the animals from both groups with regard to any investigated gene in the MSC, except for *IFN-β* (*p* < 0.01)*, IFN-γ* (*p* < 0.05) and *TNF-α* (*p* < 0.05) (Fig. 1)*.* The SRLV-infected animals displayed 0.5- and 1.0-fold lower expression of *IFN-γ* and *IFN-β,* respectively, and 3-fold higher expression of *TNF-α* compared to non-infected animals (Fig. 1).

**Fig. 2:**
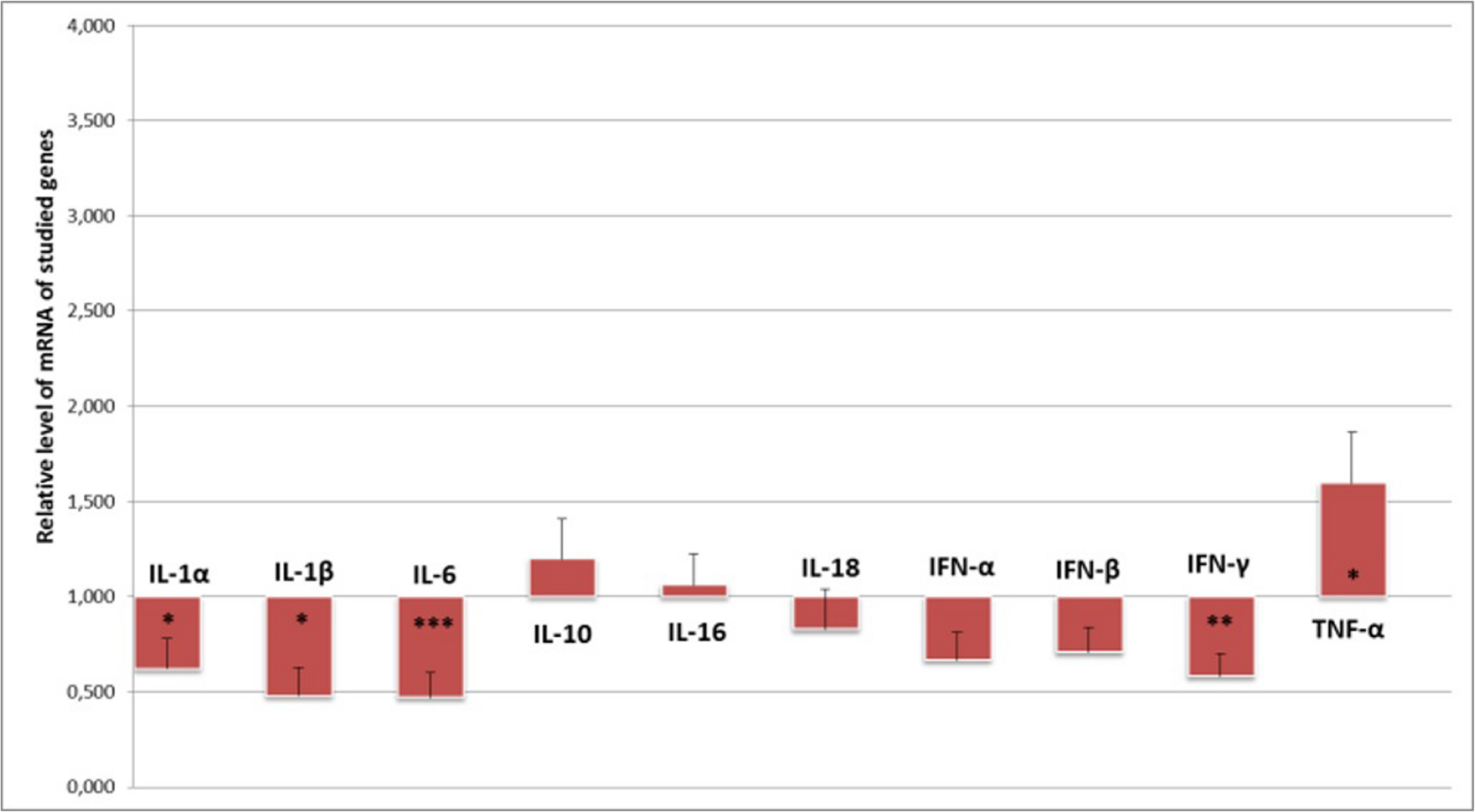
Relative expression of cytokine genes in the blood of SRLV-infected animals comparing to non-infected ones

In the blood leukocytes, the expression of *IL-1α* (*p* < 0.05), *IL-1β* (*p* < 0.01), *IL-6* (*p* < 0.001) and *IFN-γ* (p < 0.01) (Fig. 2) was decreased as a response to the presence of SRLV, all of which demonstrated 0.5-fold lower expression in SRLV-infected animals compared to non-infected ones. *TNF-α* was the only gene to display increased expression in infected animals (1.5-fold; p < 0.05) (Fig. 2). No *IL-2, IL-4* and *IL-12* transcripts were found, in the MSC or leukocytes of the infected and non-infected animals.

### Expression of selected cytokines at the protein level

**Fig. 3:**
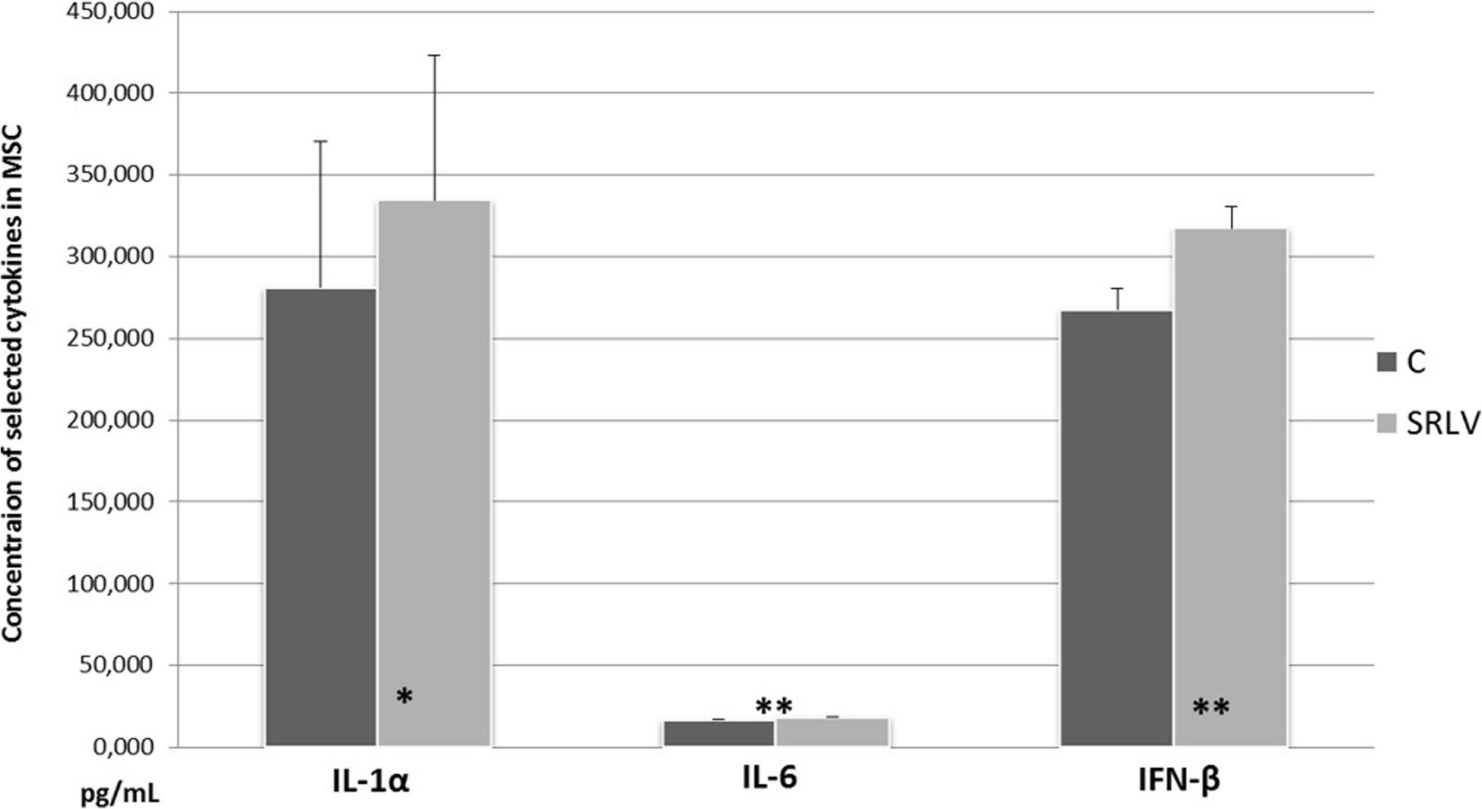
Protein concentrations of cytokines in the MSC on SRLV-infected and non-infected (C) animals

No IL-1β and IFN-γ proteins were detected in MSC of infected or non-infected animals. In addition, TNF-α was detected only at the beginning of the lactation period in both groups of animals (data not shown). The concentration of IL-6 (Fig. 3) was at the limit of detection; however significantly higher levels were observed in SRLV-infected animals (*p* < 0.02). IL-1α (*p* < 0.05) and IFN-β (*p* < 0.01) levels were also found to be elevated in the MSC of the SRLV-infected group (Fig. 3).

**Fig. 4:**
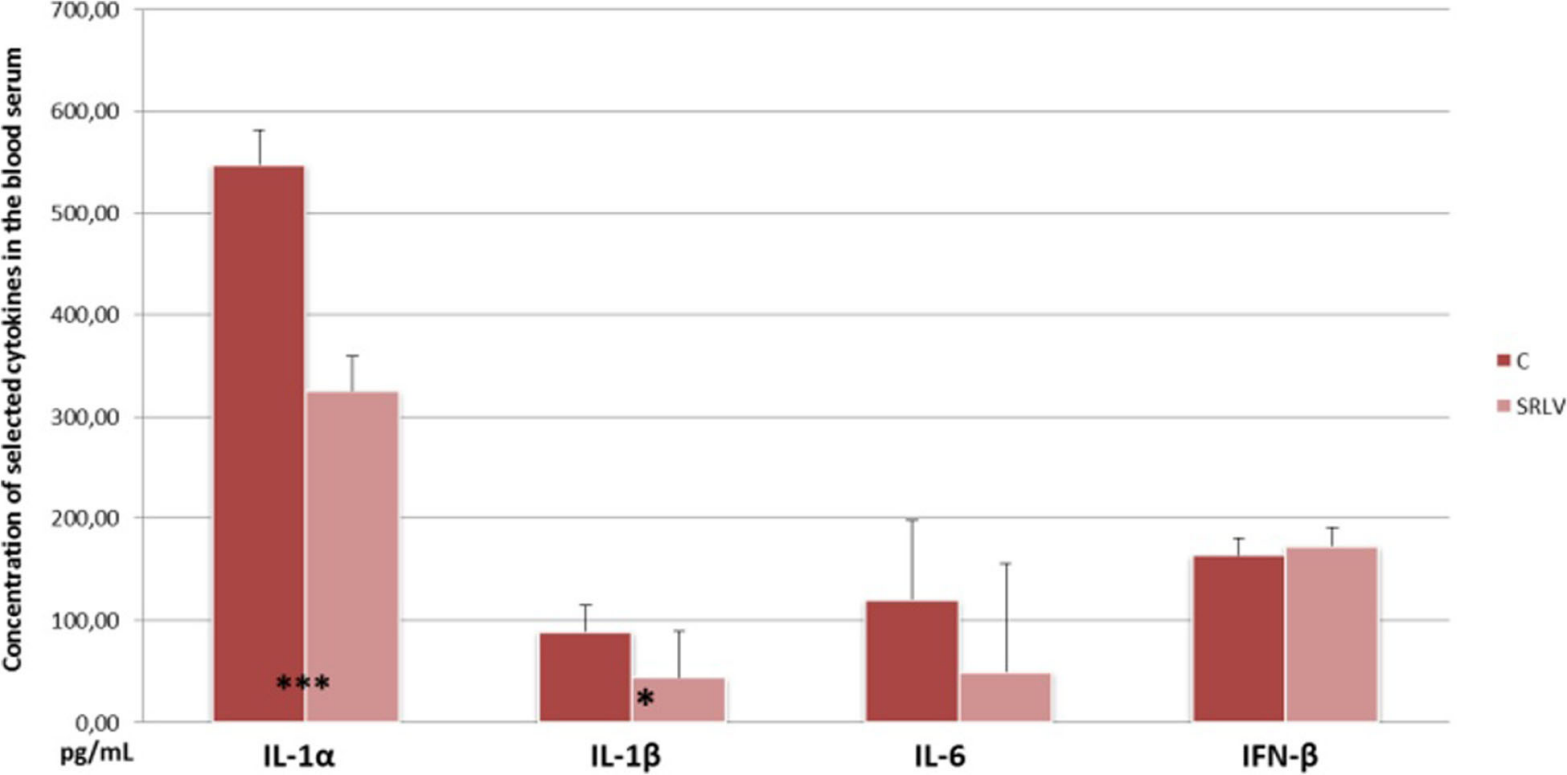
Protein concentrations of cytokines in the blood sera of SRLV-infected and non-infected (C) animals

Neither the IFN-γ and TNF-α proteins were observed in the serum of infected nor non-infected animals. No differences in IFN-β concentrations were found between the groups (*p* < 0.05) (Fig. 4); however, IL-1α (*p* < 0.001) and IL-1β (*p* < 0.05) concentrations were significantly lower in the sera of infected animals compared to non-infected ones (Fig. 4). No differences in IL-6 concentrations, were statistically confirmed (Fig. 4).

## Discussion

Deregulated cytokine responses during SRLV infection have been confirmed several times, but mainly in experimentally-infected goats [8–12] or in vitro studies [13]. Jarczak et al., [7] reported a complex analysis of the interaction between SRLV and the host immune response of naturally infected goats with regard to cytokine expression, while Reczynska et al. [14, 15] discussed the expression of acute phase proteins and cathelicidin genes during natural SRLV infection. However, relatively few in vivo studies have been performed on naturally-infected goats, or complex investigations of the gene and protein expression of cytokines, especially in the MSC and blood of infected animals.

MSC is a desirable material for non-invasive studies on the mammary gland without harm to the animals [16]. The results obtained from this kind of study provide a greater insight into the processes occurring in the udder, especially those associated with the immune response and antimicrobial defense. As goat MSC contains mainly macrophages, neutrophils and exfoliated epithelial cells [17] it is possible to obtain information on the molecular level changes occurring inside these cells following SRLV infection without the need for invasive methods.

The main finding of our study was that *TNF-α* appears to be the only cytokine gene whose transcription, i.e. mRNA level, is elevated in the MSC and blood leukocytes in SRLV-infected goats. TNF-α plays a key role in the inflammatory response, the degree of which depends on the quantity and intensity of its secretion. TNF-α stimulates the activity of monocytes and macrophages, enhances their cytotoxicity and activates the production of several proteins (IL-1, IL-6, granulocyte colony stimulating factor 3 (G-CSF), granulocyte macrophage colony-stimulating factor (GM-CSF), nerve growth factor (NGF), platelet-derived growth factor (PDGF) and epidermal growth factor (EGF)) [18]. However, according to our present results, protein expression did not correspond with mRNA expression: in both groups, the presence of *TNF-α* mRNA in MSC was detected only from the beginning of lactation until day 30, suggesting that the expression of TNF-α in MSC may be associated with the burden of goat metabolism during the perinatal period. Till the peak of lactation, mammary gland is subject to a range of dynamic processes associated with the intensive development of glandular tissue and increasing milk production. Similarly, TNF-α was not observed in blood serum throughout the whole lactation, either in infected nor non-infected animals.

TNF-α is responsible for the maturation of monocytes to macrophages, an essential stage in SRLV replication, and its increased expression might mean that SRLV uses this protein for self-replication and eventual colonization of the host organism. However, it cannot be ruled out that several post-transcriptional or post-translational modifications not related to the effect of the virus (lack of protein in biological material derived from both infected and non-infected goats) may be responsible for the fact that TNF-α protein was absent from the MSC and the blood serum; it is therefore not certain that SRLV, can use the mechanism in the same way as HIV, with the aim to colonizing the host.

Similar to the absence of TNF-α from the sera of all the animals tested in the present study, Lahtolainen et al. [19] also reported a lack of TNF-α in bovine serum. Lechner et al. [20] also noted that *TNF-α* did not appear to be elevated in animals infected with SRLV, nor was the protein found in the culture supernatants; the authors conclude that SRLV did not play a direct role in the regulation of TNF-α expression during caprine arthritis. A further study [8] examining the influence of SRLV infection on *TNF-α* gene expression found the level of *TNF-α* mRNA to be reduced in macrophages seven days after experimental infection compared to mock-infected control cells. Elsewhere, in a studies of the joints of goats with clinical arthritis, *TNF-α* mRNA was detected in infected synovium as early as six days after infection, mostly in areas infiltrated by mononuclear cells; in addition, the *TNF-α* mRNA level in the cells of the synovial membranes of goats with clinical arthritis was lower than in those infected intra-carpally 12 days post infection [8]. Unfortunately, the effect of SRLV on the regulation mRNA translation in the host is unknown, and we can only assume that the translation of TNF-α might be inhibited by one of the strategies developed by viruses.

In addition, the mRNA levels of all the studied interferons (α, β and γ) were found to be decreased in the MSC and the blood of SRLV-infected goats, both those experiencing natural and long-term infection; this may suggest that their expression was deregulated somehow by the virus. In the case of IFN-β, the protein was found to be present both in MSC and the serum however, while the mRNA expression was downregulated in the MSC, high levels of protein were observed. In addition, in the serum, no difference in protein concentration was observed between SRLV and non-infected animals. Interferons play the main role in antiviral immunity, being a group of cytokines produced and released in response to viral infections. Their synthesis is supported by several other cytokines such as IL-1, IL-2 and TNF-α. Interferons exert an indirect influence on the cell, inducing antiviral factors thorough various pathways such as the activation of protein kinase R or the *Mx* gene [21]. When the prevention of the infection as well as the induction of infected cells apoptosis are not possible, interferons may additionally influence the immune system by increasing the cytotoxic activity of lymphocyte Tc and NK cells, initiating the function of macrophages, and phagocytosis or stimulating the production of other cytokines [18].

Our results concerning *IFN-γ* gene expression did not confirm those of previous studies on SRLV-infected goats presented in the literature. An experiment on early immune response using in situ hybridization showed the peak of *IFN-γ* expression at twelve days after artificial infection and only in the synovial membranes of the joints of infected animals; after this day, no *IFN-γ* mRNA was detected in this part of the joints with subclinical or mild clinical arthritis. This may mean that SRLV stimulates local *IFN-γ* expression or vice versa only in the early phase of the disease [8]. In another study carried out on the subscapular lymph nodes of goats, *IFN-γ* expression was also confirmed, eight years after experimental infection with a molecular clone of SRLV [11]. However, as the goat with the highest provirus and viral RNA showed 3- to 8-fold higher levels of *IFN-γ* mRNA in comparison to the other four goats, these findings contradict our results at the mRNA level, even though it appeared that interferon gamma plays a role in the defense against SRLV [10]. On the other hand, IFN-γ has been described as an essential cytokine for the activation of monocytes into mature macrophages, which makes it a trigger for increased expression of SRLV in infected cells. This IFN-γ - induced activation of virus in monocytes can lead to viral replication in infected hosts being converted from a latent state to a high-level one [22].

Increased interferon genes expression is probably observed only at the beginning of infection, in the first phase of the immune response. This cannot only serve as the main defense against the virus but it can also provide a helpful way for the virus to infect the whole body. Goats with long-term SRLV infection demonstrated a deficiency in *IFNs* mRNA expression, both in MSC and the blood. Our protein analysis found that down-regulation of gene expression did not necessarily correspond with the protein concentrations, but unfortunately, no information comparable results exist regarding chronic SRLV processes in goats. Our present findings cannot unequivocally confirm that SRLV has an inhibitory effect on IFNs production, manifested as down-regulation of mRNA or protein synthesis. It was proved that viruses have developed several mechanisms to block the expression of INFs or inhibits their activity protect the agent from the host antiviral defense [21].

Although no information exists about the ability of retroviruses to inhibit IFN production, this activity has been observed among RNA viruses like influenza A or rotaviruses, and could also concern SRLV. Further analysis is needed to identify the mechanism causing increased production of IFN-β protein despite down-regulation of its gene expression. It is possible that SRLV influences the epigenetic phenomena regulating the expression of IFN at different levels.

Little is known of the expression of IL-1α and IL-1β in MSC. They are secreted proteins which are produced by activated macrophages, and their expression is typically observed in serum or plasma. IL-1α is present in MSC throughout the lactation period, but at low concentrations, which suggest its expression is constitutive but inhibited. IL-1α and IL-1β protein was also found to be down-regulated in the SRLV-infected animals, which confirms the results regarding its gene expression. In this case, SRLV was the main factor inhibiting the gene and protein expression of IL-1α and IL-1β.

Previous studies of SRLV-infected goats appear to confirm our findings. Preliminary studies by Ravazzolo et al., [11] on animals with different levels of virus load in the lymph nodes showed changes in the relative expression of four interleukins: *IL-4*, *IL-10*, *IL-12p40* and *IL-16*. Although the expression of those cytokines was found to be 2- to 11-fold higher in all samples from the goat with the highest provirus and viral RNA load in comparison to the others, the *IL-4* expression varied widely between the goats, without any correlation with the viral load; however, IL-16, a proinflammatory cytokine produced by a variety of cells including macrophages, was found to display increased expression (mRNA and protein level) in SRLV-infected goat blood. Their findings regarding IL-10 and IL-16 expression are confirmed by our own.

Sharmila et al. report higher expression of IL-16 in SRLV-infected synovial membrane cells in comparison to uninfected control cells [12] as well as an increased level of *IL-16* RNA in the synovial fluid, serum and supernatants of the PBMC cultures compared to controls. They suggest that the cytokine may be constitutively expressed at low concentration in normal, uninfected PBMCs and synovial membrane cells. Higher production of IL-16 during SRLV infection could be responsible for the increased lymphoid cell infiltrations observed in arthritic joints and other tissues.

The observed down-regulation of *IL-1α*, *IL-1β* and *IL-6* mRNA in the blood of infected animals suggests that SRLV may influence their regulation; down-regulation of IL-1α and IL-1β can lead to impairment of the immune system, manifesting in abnormal production of IL-2, IL-6 or IFN-γ, which was observed in this study. Down-regulation of IL-6 may be caused by the direct or indirect activity of IL-1α. IL-6 plays many roles in the immune system, the most important ones being the activation of lymphocytes T and B and the induction of the acute-phase reaction.

One of the first indicators of IL-6 activity is the rapid growth of the acute-phase protein production. However, no differences in C reactive protein (CRP) level in the serum were found between non-infected and SRLV-infected animals, with the results being within the normal range for adult goats, i.e. not greater than 0.51 mg/L (data not published): the upper limit is regarded as 5.0 mg/L [23]. These findings suggest that no inflammatory reaction was present within either the SRLV and non-infected hosts. They are also in line with the results of previous studies [15] and a review by Kumar et al. [24] where no increased level of CRP was observed during viral infections. They also confirm that CRP is not an acute phase protein in goat [13, 14].

In summary, our results demonstrate that natural, chronic, long-lasting SRLV infection has an influence on the expression of some genes and proteins. Several cytokines may have impaired expression under the effect of the virus, which can further result in disorders of the immune system. Our results are consistent with those obtained by Reczyńska et al. [13] regarding the expression of acute phase proteins. They also concluded that the SRLV might evade, suppress or modify the immune system. Increased expression of the transcript of *TNF-α* could serve as evidence of the virus achieving its own goals to colonize the organism; however, this is contradicted by the analysis of protein concentration. Nevertheless, our findings clearly indicate that SRLV-infected goats demonstrate different pattern of expression of several cytokines, at the gene and protein level, in both MSC and blood in comparison to healthy, non-infected animals.

## Conclusions

SRLV, a retrovirus that causes chronic and often subclinical infections, can influence the immune system of infected animals, as evidenced by changes in the expression of certain cytokines at the gene and protein level. Decreased expression of IL-1α, IL-1β and IL-6 protein suggests the immune system of infected goats is impaired, preventing them from fighting the disease. In the MSC, increased concentrations of IL-1α and IFN-β were observed in infected goats, suggesting that these cytokines play roles in the defense against the virus. In the case of blood, only IFN-β showed an increased concentration in infected goats, indicating that it also serves a defensive purpose throughout the body.

The differences observed between gene and protein expression could be interpreted as SRLV exerting a selective effect in the mechanisms regulating this expression. It may also suggest the presence of several post-transcriptional or post-translational modifications, or protein degradation processes, not connected with the effect of the virus. Further complex analyses are therefore required aimed at confirming the expression of the genes at the protein level.

Further studies are also needed to explain the actual influence of SRLV on the functioning of the immune system. The effect of SRLV on the host mRNA translational regulation is still unknown. We can only assume that translation might be inhibited by one of the strategies developed by mammalian viruses; these can range from downregulating the expression of receptors, signaling receptors, transcription factors or signaling enzymes; downregulating the proteins associated with the regulation of transcription. In addition, certain post-transcriptional and post-translational modifications can also influence the regulation of gene and protein expression, as well as and epigenetic modifications and chromatin remodeling. Epigenetic studies could be a purposeful continuation of this study, as these would provide a clearer insight into the mechanisms employed by SRLV to regulate the expression of genes and proteins by the host.

## Materials and methods

### Animals

26 Polish White Improved (PWI) and Polish Fawn Improved (PFI) dairy goats in their second to sixth lactation were included in the study. Animals were maintained on the Experimental Farm at the Institute of Genetics and Animal Breeding in Jastrzębiec, near Warsaw, Poland. The written permission about the use of the goats in this study, has been issued by the owner of the herd. The goats were kept in loose housing, and fed according to the INRA system [25]; milking was done mechanically twice a day. The experiment was conducted throughout the entire lactation period. The goats were divided by breed and parity into two groups: non-infected (*N* = 13), and SRLV naturally infected (N = 13) animals. Each group consisted of eight PWI and five PFI goats. Seven of them (4-PWI and 3-PFI) were in the older age class (third or more lactation, with finished somatic growth) and the next six (4-WPI and 2-PFI) were in their second lactation. Goats remained in the herd at the end of the experiment. They have not been euthanized.

Animals for both groups were selected on the basis of the results of at least two serological ELISA tests (IDEXX CAE/MVV Total Ab Screening Test; INC Flow Titertek Multiscan Plus Mk11, Lab systems, Finland) [26] conducted not less than six months apart. To identify possible new infection in the control group, The animals were serologically examined in December and then, to identify possible new infection in the control group, in June. The presence of the virus in this herd was confirmed both by its isolation and serological investigations carried out every year for the last 20 years [27–30]. Non-infected and infected animals were kept separate. The non-infected animals were milked first to avoid infection via milking equipment. The animals remained under the constant care of a veterinarian (employee of the Institute), who assessed the possible occurrence of infection and clinical signs of any other diseases. Furthermore, at the same time each goat was clinically examined by the certified specialist (Diplomate of the European College of Small Ruminant Health Management – coauthor JK). Irrespective of serological status, all goats in the study were asymptomatic. Furthermore, goats were tested for the presence of the virus with the use of real-time PCR method. There were no positive results observed in the animals from both groups. Prior confirmation of infection in animals from the SRLV-infected group based on the antibody levels indicates that the virus might be silent in them.

### Milk and blood samples

The milk samples were taken on days 7, 30, 120 and 240 of lactation. Each foremilk sample from all the animals was taken just before the morning milking and was examined for the presence of pathogenic bacteria according to the Malicki and Binek method [31]. Columbia agar supplemented with 5% sheep blood and MacConkey agar (BioMérieux, France) were used for bacterial culture, to identify the pathogens. Both media were inoculated with 100 μL of specimens. Plates were incubated at 37 °C for 48 h. For the confirmation of the bacteria species Vitek2 equipment was used (BioMérieux, France). To exclude pathological factors other than SRLV, only pathogen-free animals were used for the study. All milk samples were free from pathogenic bacteria. Furthermore, 64% of milk samples were free of any bacteria; however, in some cases environmental bacteria such as *Staphylococcus caprae* (19,13%), *Staphylococcus simulans* (6,74%), and *Staphylococcus epidermidis* (3%) were identified (in both groups of animals and at similar levels – data not published).

Furthermore, milk was analyzed on the somatic cell count (SCC) using an IBCm instrument (Bentley, MN, USA). No differences were observed in SCC in SRLV-infected and non-infected animals (data not published); the level of SCC in the milk of animals from both groups did not indicate on sub-clinical inflammations of the mammary gland.

Next, one liter of milk was obtained during morning milking from each goat for the isolation of somatic cells. Milk was firstly centrifuged at 1500 x g for 30 min. The somatic cell pellet was then washed with phosphate buffered saline (PBS) and centrifuged at 1100 x g for 15 min. This step was repeated twice. One ml of Trizol reagent was used to dissolve the pellet which was then stored at − 80 °C.

Whole blood samples were taken in EDTA tubes as well as into 9 mL tubes with clot-activator (Sarstedt AG & Co., Germany) by a veterinarian, on the same day as the milk sample collection. Blood for serum was then centrifuged at 3000 x g for 20 min at 4 °C. The serum was collected and frozen at − 80 °C for further analysis. The following biochemical parameters were examined in serum using an INTEGRA system:

- hepatic profile - determining albumin level (ALB), total bilirubin (BILT), alanine aminotransferase (ALAT) and aminotransferase aspartate (AST).

- renal profile - determining the level of creatinine (CRE) and total protein (TP).

- bone profile - determining the level of calcium (Ca) and alkaline phosphatase (ALP).

- the cardiac profile - determining the level of creatinine kinase (CK) and gamma glutyltranspeptidase (GGT) as well as electrolytes (chlorides - Cl, sodium - Na, potassium - K, magnesium - Mg, iron - Fe).

- pancreatic profile - determining the level of lipase (LIP) and glucose (GLU).

- lipid profile - determining the level of cholesterol (CHOL) in this fraction high density lipoprotein (HDL) and low density lipoprotein (LDL) and also triglycerides (TRI).

There were no differences in the biochemical parameters in serum between SRLV-infected and non-infected animal, except for calcium (data not published).

### RNA isolation and cDNA synthesis

Total RNA was isolated from MSC using a Pure Link RNA Mini Kit (Ambion, USA) according to the manufacturer’s instructions. The isolation was supported by a DNAse digestion to remove genomic DNA using a PureLinkDNase Set (Invitrogen, USA). The total RNA from the blood samples was isolated using a NucleoSpin RNA Blood kit (Macherey-Nagel, Germany). Erythrocytes were degraded with the use of Red Blood Cell Lysis Buffer (Roche, Switzerland). The isolated RNA has been subjected to a qualitative and quantitative assessment using a NanoDrop spectrophotometer (NanoDrop, USA) and Bioanalyzer 2100 (Agilent Technologies, France). Information about the mean values of RNA is shown in Table 1.

**Table 1:** Mean values of RNA extracted from the MSC and whole blood of goats

Mean values	MSC^a^	Blood
Quantity [ng/μL]	167.88	69.41
A260/A280 ratio	1.98	1.82
A260/A230 ratio	1.44	1.20
RIN^#^	5.25	6.0

^a^MSC – milk somatic cells; ^#^RIN – RNA Integrity Number

Following this, 1 μg of RNA was reverse transcribed into cDNA using a Transcriptor First Strand cDNA Synthesis Kit (Roche, Switzerland). cDNA samples were diluted to a volume of 50 ng and used in Real-time PCR analysis.

### Primers

Primers for housekeeping genes used in this study (cyclophilin A (*PPIA)* for MSC, protein zeta (*YWHAZ)* for blood samples) have been previously described [31]. The sequences of primers for the studied genes (*IL-1α, IL-1β, IL-2, IL-4, IL-6, IL-10. IL-12, IL-16, IL-18, IFNα, IFNβ, IFNγ,* and *TNFα*) were designed on the basis of *Capra hircus* or *Bos taurus* sequences available in the NCBI data base using Primer BLAST Software [32] or taken from the literature [9, 11, 33]. The forward and reverse primers of all the studied and reference genes were designed in two separate exons [34]. All primer sequences for caprine cytokines are shown in Table 2. The specificity of primers was tested using the caprine pooled cDNA in preliminary PCR analysis. For *IL-2*, *IL-4* and *IL-12* two different pairs of primers were tested.

**Table 2:** The primer sequences used for the studied cytokines and reference genes

Protein	Gene	Primer sequence	Position	Product size (bp)	Accession No. from GenBank	Reference
Interleukin 1 alpha	*IL-1α*	F:TGAACGACGCCCTCAATCAA R: CTTGCCATGTGCACCAGTTT	423-442 789-761	358	D63350.1	designed
Interleukin 1 beta	*IL-1β*	F: GCAGCTGGAGGAAGTAGACC r: TGGCTTTCTTTAGGGAGAGAGG	585-604 816-795	232	D63351.1	designed
Interleukin 2	*IL-2*	F: ATGTACCAGATACCACTCTTGTCTT R: TCAAGTCATTGTTGAGTAGAT	22–46 489–469	468	AY603404.1	(9)
Interleukin 4	*IL-4*	F:TAGCTTCTCCTGATAAACTA R: ATGAGTTATAAATATATAAATA	1–20 535–514	535	U34273.1	(9)
Interleukin 6	*IL-6*	F: TCTTCACAAGCGCCTTCAGT R: CTGCTTGGGGTGGTGTCATT	11-30 131-112	121	D86569.1	designed
Interleukin 10	*IL-10*	F: CGGCGCTGTCATCGTTTT R: TCTTGGAGCATATTGAAGACTCTCTTC	364-381 446-420	83	DQ837159.1	(11)
Interleukin 12	*IL-12*	F: CACCAAAGATAAAACCAGCACAGT R: GTCTTTCCAGAAGCCAGACAATG	180-203 305-283	126	AY603407.1	(11)
Interleukin 16	*IL-16*	F: AAAAGACCTCTGCGGGACTG R: TCAGGCAACGCCTTGATGAT	101-120 314-295	214	AF481158.1	designed
Interleukin 18	*IL-18*	F: TCCTAAGAAGCTATTGAGCACAGGC R: ATTTTAATATCTAGTCTGGTTTTG	9-33 628-604	611	AY605263.1	(33)
Interferon alpha	*IFN-α*	F: CACCTTCCAGCTCTTCAGCA R: GTCAGTGAGCTGCTGATCCA	258-277 354-335	97	FJ959074.1	designed
Interferon beta	*IFN-β*	F: ACAGCAGTTCCGGAAGGAAG R: TCGGTCGTGTCTCCCATAGT	204-223 416-397	213	JX458085.1	designed
Interferon gamma	*IFN-γ*	F: TAGCTAAGGGTGGGCCTCTTTTCTCA R:TGCAGGCAGGAGAACCATTACATTGA	189–214 573-548	385	AY603405.1	(9)
Tumor NecrosisFactoralpha	*TNF-α*	F: AGAAGGGAGATCGCCTCAGT R: AGAAGGGGATGAGGAGGGTC	488-507 659-640	172	X14828.1	designed
Cyclophilin A	*PPIA*	F:GGATTTATGTGCCAGGGTGGTGA R: CAAGATGCCAGGACCTGTATG	186-208 305-285	120	AY 247029.1	(36)
Zeta polypeptide	*YWHAZ*	F:AGACGGAAGGTGCTGAGAAA R:CGTTGGGGATCAAGAACTTT	290-309 412-393	122	BC102382.1	(36)

### Real-time PCR

The levels of transcripts were measured using real-time PCR method in a Light Cycler System (Roche, Switzerland). Reactions were carried out in duplicates with a non-template control, on 384-well optical reaction plates. Received gene expression profiles were subjected to normalization with reference genes. All data, including the raw cycle threshold (Ct), which was obtained using Light Cycler 480 Software, were used for the comparative Ct method [35]. The information about efficiencies, together with all information about the used primers is shown in Table 2. The details of the methodology has been previously described in Jarczak et al., [36].

### Protein extraction and ELISA tests

Detection of several cytokines (IL-1α, IL-1β, IL-6, IFN-β, IFN-γ and TNF-α) at the protein level was carried out in the serum and cell extracts coming from MSC using ELISA kits containing specific antibodies. Whole blood collected in tubes with a coagulation activator was centrifuged at 3000 x g for 20 min at 4 °C. The serum was collected and frozen at − 80 °C for further analysis. Protein extracts from MSC were homogenized in 1 mL of PBS and stored overnight at − 20 °C. After two freeze-thaw cycles the homogenates were centrifuged at 5000 x g for 5 min at 4 °C. The supernatant was aliquated and stored at − 80 °C. The procedure was supported with a protease inhibitor (Roche, Switzerland). The mean values of protein quantity are shown in Table 3.

**Table 3:** Mean values of protein extracted from MSC or measured in the serum

Mean values	MSC^a^	Serum
Quantity	2.28 mg/mL	56.68 mg/mL

^a^MSC – milk somatic cells

The quantities of the extracted protein was assessed using a spectrophotometer according to Bradford method [37]. The selected cytokines were assayed quantitatively by ELISA according to the manufacturer’s instructions (Wuhan EIAab, China; Kamiya Biomedical, USA). Data were obtained as the mean of duplicate readings for each standard, control and samples at 450 nm. The standard curve was generated, and a four parameter logistic curve-fit was created to calculate the results. Protein concentrations obtained by the ELISA tests are given in pg/mL for all studied tests.

### C reactive protein (CRP) level

Blood samples were taken in tubes containing the coagulation activator by a veterinarian,, on the same day as the milk sample collection. The serum was centrifuged and CRP level was analyzed using INTEGRA (Roche, Switzerland) apparatus.

### Statistical analysis

All variables were tested for normality of distribution and relative gene expressions were transformed into natural logarithm scale. Multi-factor analysis of variance was then performed, to compare the fixed effect of virus infection, breed, age of the animals and state of lactation. The final model did not contain the presence/absence of bacteria effect because in prion analysis no its impact on gene expression was found. A *p*-value 0.05 was considered statistically significant. Calculations were carried out using SAS/STAT software with analyze of variance method (procedure GLM).

## Data Availability

Data will be made available from the corresponding author on request.

## Abbreviations

ASFV: African Swine Fever Virus
CNS: Central nervous system
CRP: C-reactive protein
E3L: Protein E3L
EBV: Epstein-Barr Virus
EGF: Epidermal growth factor
EIAV: Equine Infectious Anemia Virus
EIF2AK2: Eukaryotic Translation Initiation Factor 2 Alpha Kinase 2
FIV: Feline Immunodeficiency Virus
G-CSF: Granulocyte- colony stimulating factor 3
GM-CSF: Granulocyte macrophage - colony stimulating factor
HIV: Human Immunodeficiency Virus
IFN: Interferon
IL: Interleukin
IκB: kinase IκB
MCP-1: Monocyte chemoattractant protein 1
MSC: Milk somatic cells
NF-κB: Nuclear factor of kappa light polypeptide gene enhancer in B cells
NGF: Nerve growth factor
NK cells: Natural killer cells
NS1: NS1 influenza protein
NSP3: Non-structural protein 3
PBMC: Peripheral blood mononuclear cells
PDGF: Platelet-derived growth factor
PFI: Polish Fawn Improved (breed of domestic goat)
PPIA: Cyclophilin A
PWI: Polish White Improved (breed of domestic goat)
RIN: RNA integrity number
SIV: Simian Immunodeficiency Virus
SRLV: Small Ruminant Lentivirus
SU: Surface protein
Th2 cells: T helper cells type 2
TNF: Tumor necrosis factor
YWHAZ: Protein zeta
